# Fasting gut hormone levels following long-term recovery from Anorexia Nervosa: A cross-sectional study

**DOI:** 10.1186/s40337-026-01763-y

**Published:** 2026-09-10

**Authors:** Theresa Kolb, Friederike Ingrid Tam, Claudia Förster Ribet, Michaela Ohme, Lina Carlotta Schlolaut, Joseph Adam King, Veit Roessner, Suzanne Lee Dickson, Carmine Maria Pariante, Alessandra Borsini, Nikolaos Perakakis, Stefan Ehrlich

**Affiliations:** 1 Division of Psychological and Social Medicine and Developmental Neurosciences, Faculty of Medicine, Technische Universität Dresden, Dresden, Germany; 2 Department of Child and Adolescent Psychiatry and Psychotherapy, University Hospital Carl Gustav Carus, Technische Universität Dresden, Dresden, Germany; 3 Institute of Neuroscience and Physiology, The Sahlgrenska Academy at the University of Gothenburg, Gothenburg, Sweden; 4 Stress, Psychiatry and Immunology Laboratory, Institute of Psychiatry, Psychology and Neuroscience, Department of Psychological Medicine, King’s College London, London, UK; 5 Department of Internal Medicine III, University Hospital Carl Gustav Carus, Technische Universität Dresden, Dresden, Germany; 6 Helmholtz Center Munich, Faculty of Medicine, Paul Langerhans Institute Dresden (PLID), University Hospital, Dresden, Germany; 7 German Center for Diabetes Research (DZD e.V.), Neuherberg, Germany; 8 German Center for Child and Adolescent Health (DZKJ), partner site Leipzig/Dresden, Dresden, Germany

**Keywords:** Anorexia Nervosa, GLP-1, GIP, Gut hormones, Oral contraceptives, Recovery

## Abstract

**Background:**

Glucagon-like peptide-1 (GLP-1), gastric inhibitory peptide (GIP), glucagon, and glicentin are gastrointestinal hormones derived from proglucagon that play crucial roles in regulating appetite and glucose homeostasis. These hormones have been examined during the acute state of anorexia nervosa (AN), with our previous study showing elevated GIP levels in the acute state which normalized following short-term weight restoration. GLP-1 and glicentin concentrations remain unchanged in the acute state, but decrease after short-term weight restoration. However, few studies have investigated whether these alterations are more than mere consequences of the severe underweight, whether they persist after long-term recovery, and whether they could therefore be considered potential trait markers.

**Methods:**

In the current study, we assessed fasting serum concentrations of GLP-1, GIP, glucagon, and glicentin in 80 female individuals who had long-term recovered from AN and 100 healthy female controls. For group comparisons, a subset of 70 controls was pairwise age-matched to the recovered patients. Participants were further stratified by oral contraceptive (OC) use.

**Results:**

No significant differences in hormone concentrations, confirmed by Bayesian statistics, were observed between controls and recovered patients, regardless of OC use, except for glucagon levels.

**Conclusion:**

These findings suggest that alterations in gut-secreted hormones associated with glucose homeostasis during the acute, malnourished phase of AN reflect state-dependent changes that do not persist after recovery. OC use in former patients with AN does not affect the concentration of these hormones during recovery.

**Supplementary information:**

The online version contains supplementary material available at https://doi.org/10.1186/s40337-026-01763-y.

## Abstract (plain language summary)

Anorexia nervosa (AN) is a serious eating disorder that can be life-threatening. People with AN restrict their food intake severely, experience intense fear of weight gain, and have a distorted body image. It most commonly affects girls and young women. People with this condition often have imbalanced levels of certain hormones that help control hunger and blood sugar. In this study, we wanted to know if these hormone imbalances are just temporary effects of being severely underweight, or if they might be long-lasting characteristics that could help explain part of the illness. We measured four different hormones in 80 women who had fully recovered from AN and compared them to 100 healthy women who had never had the condition. We also tested whether using birth control pills (which affect a variety of hormones in the body) made any difference to the results. Our findings showed that the hormone levels in recovered women were almost identical to those in healthy women. This suggests that the unusual hormone levels seen when someone is actively ill with AN are caused by the state of being underweight, rather than being a permanent characteristic. Once a person recovers and restores their weight, these hormone levels return to normal. Birth control pill use did not affect hormone levels in recovered women.

**Supplementary information:**

The online version contains supplementary material available at https://doi.org/10.1186/s40337-026-01763-y.

## Introduction

Anorexia Nervosa (AN) is a complex and life-threatening eating disorder that typically manifests during adolescence. It is characterised by severe undernutrition, disturbances in body image, and an intense fear of weight gain, predominantly affecting girls and young women [1–3]. Even though there have been some advances in therapeutic approaches, less than half of the affected individuals achieve full recovery, and AN remains one of the most lethal psychiatric conditions, with a mortality rate of approximately 5% [3, 4].

Energy restriction due to the severe undernutrition in AN leads to widespread physiological adaptations across multiple systems, including alterations in brain structure and function [5] and profound endocrine disturbances [6–8]. Among these are well-established key-regulators of appetite and energy homeostasis, such as leptin and ghrelin, which have been extensively studied in AN [9–12]. In contrast, gut-secreted hormones such as glucagon-like peptide-1 (GLP-1), glucose-dependent insulinotropic polypeptide (GIP), glicentin, and glucagon have received comparatively less attention in AN research, despite their established roles in regulating appetite and glucose homeostasis [13].

GLP-1 and GIP are incretin hormones that stimulate insulin secretion and modulate glucagon release – GLP-1 inhibits, while GIP stimulates glucagon secretion [13, 14]. Furthermore, both incretins reduce appetite via central mechanisms [15–17]. Glicentin, a co-secreted proglucagon-derived peptide, has been suggested to play roles in insulin secretion and gastrointestinal function, with emerging evidence also linking it to appetite/weight regulation in humans [18, 19]. Glucagon, best known for its role in hepatic glucose production [20] via glycogenolysis and gluconeogenesis, has been shown to suppress appetite and influence energy balance in experimental, animal, and selected human studies.

Some older studies have reported that levels of GLP-1, GIP, and glucagon are altered in acute and short-term recovery phases of AN, but findings were inconsistent, likely due to methodological differences in assays available at the time and small sample sizes [20–31]. To our knowledge, no studies have investigated glicentin in AN. In our prior work, we examined all four hormones together (i.e. including glicentin) for the first time using state-of-the-art assays to provide a broader perspective and understanding of their roles in AN [32]. We found that GIP levels were higher during the acute phase of the illness and normalized after short-term weight restoration, while GLP-1 and glicentin were not altered in acute AN but decreased after short-term weight restoration. Notably, glucagon levels remained unchanged throughout.

Whether these hormonal changes reflect temporary adaptations to undernutrition or persist beyond recovery remains unknown. To our knowledge, no previous study assessed these hormones collectively in a cohort of former AN patients who have achieved stable recovery for a substantial amount of time. However, investigating individuals who are long-term recovered from AN (recAN) can help to disentangle state-related effects from potential trait markers [4, 33].

State markers would be hormonal alterations strongly linked to the acute undernutrition in AN and normalize after weight restoration. In contrast, if disruptions in these hormones persist independently of the state of illness, they may represent trait markers. Differentiating between these state and trait characteristics is important in the clinical context. State markers guide acute treatment, while trait markers can help to identify individuals at risk for the disorder or risk for relapse and could be used to implement preventive measures.

Unlike patients in the acute phase of AN [34], oral contraceptive (OC) use is common in a significant portion of recAN, just like in healthy controls (HC). OC use may potentially influence glucose metabolism, although evidence on their metabolic impact is mixed and appears to depend on the specific formulation used [35–39]. Given the widespread use of OC, it is therefore necessary to account for OC in studies investigating hormones involved in glucose metabolism. Accordingly, in our present study, we stratified our participants into two groups corresponding to OC use.

The aim of this study was to assess fasting serum concentrations of GLP-1, GIP, glucagon and glicentin in recAN compared to HC, with stratification by OC use. This design allows us to gather evidence on whether concentrations of these hormones after long-term recovery from AN differ from those in HC, and to determine whether they represent temporary state markers of acute undernutrition or more permanent trait markers that may indicate an underlying risk factor. Further, we wanted to explore the possible influence of OC use on gut hormone profiles in this population.

## Methods

### Participants

Serum samples from 180 female participants were included in the current study: 80 recAN (age range: 14.0–29.8 years) and 100 HC (age range: 14.0–29.7 years). Participants were further stratified into groups based on OC use, coded as “^+^” (OC users) or “^−^” (non-users). Due to significant age differences between the OC^−^ groups, HC^−^ were pairwise age matched (Supplemental Material, 1.1.) to recAN^−^, resulting in 35 participants per group. However, residual age-differences remained between the OC^−^ groups and we therefore included age as a covariate in the analysis of these groups. The OC^+^ groups did not differ in age and consisted of 45 recAN^+^ and 35 HC^+^. Overall, 70 HC were included in the age-matched analyses (35 per OC group), while the whole unmatched sample was used for the supplementary analysis of the HC groups.

Both the recAN and HC groups were recruited via widespread community advertisement, such as flyers and social media posts. RecAN participants were required to have had a past diagnosis of AN and, for at least 6 months before the study, to [1] maintain a body mass index (BMI) above the 10th age percentile (if younger than 18 years) or above 18.5 kg/m^2^ (if older than 18 years) [2], menstruate, and [3] refrain from bingeing, purging, or engaging in substantial restrictive eating patterns. HC had to be of normal weight, eumenorrheic, and free of any history of mental illness, and were recruited through advertisement among middle/high school and university students. Current and past diagnoses of eating disorders and other pertinent information, including potential confounding variables (e.g. medication, comorbidities, menstrual cycle), were obtained in all participants using the expert form of the Structured Interview for Anorexia and Bulimia Nervosa (SIAB-EX) [40], supplemented with an in-house semi-structured interview. The Mini International Neuropsychiatric Interview [41] was used to screen for psychiatric conditions. Participants of both groups were excluded if they had a history of any of the following diagnoses: organic brain syndrome, schizophrenia, substance dependence, psychosis not otherwise specified, bipolar disorder, bulimia nervosa, or binge-eating disorder. Further exclusion criteria for all participants were an IQ below 85; current substance abuse; inflammatory, neurologic, or metabolic illness; chronic medical or neurological illness that could affect appetite, eating behaviour or body weight; clinically relevant anemia; pregnancy or breast feeding. Use of any psychotropic medication within 4 weeks before the study was an additional exclusion criterion for both groups, with the exception of selective serotonin reuptake inhibitors in the recAN group.

The mean duration since weight normalization in the recAN group was 53.3 ± 39.1 months. In the recAN group, 58/80 used to be of the restrictive AN subtype, while 22/80 were of the binge/purge AN subtype at the time of treatment. Of the recAN participants, 31/80 had psychiatric comorbidities in the past, and 7/80 were treated with selective serotonin reuptake inhibitors. Regarding ethnicity, all recAN participants (*N* = 80) identified as “European heritage”. In the HC group (*N* = 100), 98 identified as “European heritage”, one identified as “African heritage”, and one identified as “other”.

All protocols received ethical approval by the local Institutional Review Board, and all participants (or if underage, their guardians) gave written informed consent. The study was performed in accordance with the ethical standards as laid down in the 1964 Declaration of Helsinki and its later amendments.

Study data were collected and managed using the secure, web-based electronic data capture tool REDCap (Research Electronic Data Capture) [42, 43].

### Clinical measures

In addition to the evaluation with the SIAB-EX [40], eating disorder-specific psychopathology was assessed with the German version of the self-report questionnaire Eating Disorder Inventory-2 (EDI-2) [44]. Depressive symptoms were explored using the German version of the Beck Depression Inventory-II (BDI-II), while general levels of psychopathology and anxiety symptoms were evaluated using the Symptom-Checklist-90 Revised (SCL-90-R) [45]. The intelligence quotient (IQ) was estimated with short versions of the German adaptation of the Wechsler Adult Intelligence Scale [46] or, for study participants aged 15 years or younger, the Wechsler Intelligence Scale for Children (Hamburg-Wechsler Intelligenztest für Kinder IV) [47]. In addition to the BMI, the age-corrected BMI standard deviation score (BMI-SDS) was calculated [48, 49].

### Procedure

Venous blood samples were collected between 7 and 9 a.m. following an overnight fast. To obtain blood serum for assessment of glucose, insulin, GIP, GLP-1, glicentin, and glucagon, samples were left to clot for 30 min at 6–8 °C and then centrifuged at 2500 x *g* for 15 min at 5 °C. To yield plasma for leptin assessment, aprotinin was added during blood sampling to inhibit protein degradation by serine proteases. These samples were immediately centrifuged under the same conditions (2500 x *g*, 15 min, 5 °C). All samples were aliquoted immediately after centrifugation and stored at -80 °C until analysis.

Serum concentrations of insulin (Mercodia Cat# 10-1113-01, RRID: AB_2877672), GIP (Mercodia Cat# 10-1258-01, RRID: AB_2895085), GLP-1 (Mercodia Cat# 10-1278-01, RRID: AB_2892202), glicentin (Mercodia Cat# 10-1273-01, RRID: AB_2884906), and glucagon (Mercodia Cat# 10-1271-01, RRID: AB_2737304) were quantified using commercially available enzyme-linked immunosorbent assays (ELISAs). Plasma leptin levels were determined using the Leptin Human ELISA (BioVendor Cat# RD191001100, RRID: AB_3696585;Clinical Range; Brno, Czech Republic). Serum glucose concentrations were measured using a Super GL compact analyzer (Dr. Müller Gerätebau GmbH).

### Statistical analysis

Statistical analyses were conducted using R (version 4.2.1) and JASP [50]. Descriptive statistics were computed for demographic, clinical, and blood parameters across all groups.

Statistical significance was set at *p*<.05. Where appropriate, *p*-values were adjusted for multiple comparisons using the False Discovery Rate (FDR) correction method by Benjamini and Hochberg (1995) [51]. To assess group differences in demographic and clinical variables between recAN^−^ vs. HC^−^, as well as between recAN^+^ vs. HC^+^, independent-samples t-tests (and in rare cases of non-normal data distribution Mann-Whitney U tests) were performed. Between recAN^−^ and HC^−^, as well as in supplemental analyses between HC^−^ and HC^+^, analyses of covariance (ANCOVAs) were conducted on variables that showed significant group effects to assess remaining age differences.

Most blood parameter concentrations, with the exception of glucose, showed skewed distributions across groups. To normalize distributions, a logarithmic transformation (log_10_) was applied to insulin, GLP-1, GIP, glucagon, and glicentin values. Outliers were identified as values exceeding ± 3 standard deviations (SD) from the group mean and were winsorized to the 3 SD boundary for the respective variable [52].

Due to residual age differences between the recAN^−^ and HC^−^ groups, comparisons of blood parameters between these groups were performed using ANCOVAs, with age as a covariate. For comparisons between recAN^+^ and HC^+^, independent-samples *t*-tests were used to compare blood parameters. In cases where frequentist analyses indicated no significant group differences, Bayesian *t*-tests and Bayesian ANCOVAs were conducted post hoc to quantify evidence in favour of the null hypothesis (i.e., no group difference). Additionally, supplementary group comparisons of the log-transformed blood parameters between HC^−^ and HC^+^ were performed using ANCOVAs to control for age differences (Supplemental Table 5).

In exploratory analyses, associations between hormone concentrations and the number of months since recovery were examined in recAN^−^ using Spearman rank-order correlations (Supplemental Table 6).

## Results

### Demographic, anthropometric and symptom variables

Demographic and clinical characteristics are summarized in Table 1 (OC^−^ groups) and Table 2 (OC^+^ groups). Across both OC^−^ and OC^+^ groups, BMI, BMI-SDS, and IQ scores did not differ between recAN and HC, but recAN participants were older than HC in both OC groups. As expected, recAN participants had a significantly lower minimal lifetime BMI. Regardless of OC use, recAN also showed somewhat higher levels of psychopathology (as indicated by significantly higher EDI-2, BDI-II, and SCL-90-R scores) compared to HC. In the OC^−^ groups, differences persisted after controlling for age (Supplemental Table 1). In addition, leptin levels were decreased in recAN^+^ compared to HC^+^, but did not differ between recAN^−^ and HC^−^.

**Table 1:** Groups without oral contraceptive use (OC^−^): Demographic and clinical characteristics

	*HC* ^−^		*recAN* ^−^		Group Comparison
	*N*	Mean ± SD Median (IQR)	*N*	Mean ± SD Median (IQR)	*p*-value
**Age**	35	16.30 (2.20)	35	23.00 (4.65)	< 0.001**
**BMI (kg/m** ^**2**^ **)**	35	21.27 ± 2.09	35	22.57 ± 3.85	0.801
**BMI-SDS**	35	-0.07 ± 0.75	35	-0.38 ± 0.71	0.072
**Minimal Lifetime BMI (kg/m** ^**2**^ **)**	33	20.25 ± 1.84	35	14.14 ± 1.69	< 0.001**
**IQ**	34	110.53 ± 8.92	35	111.93 ± 10.19	0.546
**Months since Recovery**	35	-	35	36 (64.5)	-
**BDI-II**	32	3.00 (5.00)	33	9 (13.00)	0.004*
**EDI-2**	32	134.50 (38.25)	34	169.5 (57.41)	< 0.001**
**SCL-90-R**	32	22.50 (22.00)	34	31 (42.00)	0.030*
**Leptin (ng/ml)**	31	9.00 (8.82)	32	7.1 (4.89)	0.114

Note. BDI-II=Beck Depression Inventory-II; BMI=body mass index; BMI-SDS=body mass index standard deviation score; EDI-2 = Eating Disorder Inventory-2; HC=healthy controls; IQ=intelligence quotient; kg/m²=kilograms per meter squared; ng/ml=nanograms per millilitre; recAN=long-term recovered anorexia nervosa patients; SCL-90-R=Symptom Checklist-90-Revised. Mean±standard deviation is reported for normally distributed parameters; median (interquartile range) is reported for non-normally distributed data. Group comparisons were performed using Student’s t-test or Mann–Whitney U test, as appropriate. Asterisks indicate statistical significance (uncorrected, two-tailed): *p*<.01 (**), *p*<.05 (*)

**Table 2:** Groups with oral contraceptive use (OC^+^): Demographic and clinical characteristics

	*HC* ^+^		*recAN* ^+^		Group Comparison
	*N*	Mean ± SD Median (IQR)	*N*	Mean ± SD Median (IQR)	*p*-value
**Age**	35	22.9 (4.45)	45	22.3 (4.6)	0.488
**BMI (kg/m** ^**2**^ **)**	35	21.92 ± 2.43	45	21.23 ± 2.04	0.182
**BMI-SDS**	35	-0.18 ± 0.68	45	-0.37 ± 0.58	0.188
**Minimal Lifetime BMI (kg/m** ^**2**^ **)**	33	20.11 ± 1.68	45	14.47 ± 1.52	< 0.001**
**IQ**	35	110.23 ± 9.56	45	110.8 ± 10.64	0.801
**Months since Recovery**	35	-	45	40 (46)	-
**BDI-II**	29	1 (3)	43	4 (12.5)	0.001**
**EDI-2**	28	124.5 (22.74)	42	147 (60.75)	0.003**
**SCL-90-R**	28	9 (12.75)	42	23.5 (57.5)	0.003**
**Leptin (ng/ml)**	29	11.85 (11.6)	42	8.4 (5.07)	0.020*

Note. BDI-II=Beck Depression Inventory-II; BMI=body mass index; BMI-SDS=body mass index standard deviation score; EDI-2 = Eating Disorder Inventory-2; HC=healthy controls; IQ=intelligence quotient; kg/m²=kilograms per meter squared; ng/ml=nanograms per millilitre; recAN=long-term recovered anorexia nervosa patients; SCL-90-R=Symptom Checklist-90-Revised. Mean±standard deviation is reported for normally distributed parameters; median (interquartile range) is reported for non-normally distributed data. Group comparisons were performed using Student’s t-test or Mann–Whitney U test, as appropriate. Asterisks indicate statistical significance (two-tailed): *p*<.01 (**), *p*<.05 (*)

Regarding HC groups, HC^+^ were significantly older than HC^−^ before age-matching. They did not differ in other demographic parameters, but HC^−^ displayed significantly higher scores in psychopathological measures (Supplemental Table 2). After controlling for age, there were no differences in clinical or demographic parameters between the groups (Supplemental Table 3, Supplemental Table 5).

### Differences in parameters of glucose homeostasis and gut-secreted hormones

#### OC^−^ groups

Concentrations of the blood parameters in OC^−^ groups, presented as original values, are summarized in Table 3 alongside group comparison results after log_10_-transformation. No significant group effects were observed for any of the parameters after adjusting for age and multiple comparisons (all *p(FDR)*>0.05) (Table 3). This was verified by Bayesian statistics for glucose (*BF*_*01*_=3.861), insulin (*BF*_*01*_=7.509), GIP (*BF*_*01*_=4.013), GLP-1 (*BF*_*01*_=2.196) and glicentin (*BF*_*01*_=3.686). For glucagon (*BF*_*01*_=1.000), the uncorrected *p*-value from the frequentist approach was < 0.05, suggesting a potential group difference.

**Table 3:** Groups without oral contraceptive use (OC^−^): Blood glucose and hormone concentrations

	*HC*^−^ (*N* = 35)	*recAN*^−^ (*N* = 35)	ANCOVA	FDR	
	Mean ± SD Median (IQR)	Mean ± SD Median (IQR)	*p*	*p*	BF_01_
**Glucose (mmol/l)**	4.97 ± 0.41	5.01 ± 0.4	0.709	0.851	3.861
**Insulin (pM)**	30.94 (20.79)	28.43 (13.62)	0.525	0.851	7.509
**GIP (pM)**	5.34 (3.76)	4.81 (3.31)	0.655	0.851	4.013
**GLP-1 (pM)**	3.62 (1.37)	4.08 (1.93)	0.235	0.705	2.196
**Glicentin (pM)**	31.1 (17)	28.44 (24.03)	0.879	0.879	3.686
**Glucagon (pM)**	4.58 (2.3)	3.69 (2.44)	0.067	0.402	1.000

Note. GIP=glucose-dependent insulinotropic polypeptide; GLP-1 = glucagon-like peptide-1 (GLP-1); HC=healthy controls; mmol/l= millimoles per litre; pM=picomolar; recAN=long-term recovered anorexia nervosa patients. Mean±standard deviation is reported for normally distributed parameters; median (interquartile range) is provided for non-normally distributed data. GIP, GLP-1, glicentin, and insulin concentrations were log₁₀-transformed prior to ANCOVA. Age was included as a covariate. *P*-values were adjusted for multiple comparisons using the False Discovery Rate (FDR) method [49]. Asterisks indicate statistical significance (two-tailed): *p*<.01 (**), *p*<.05 (*). Bayes factors (*BF₀₁*) > 1 indicate increasing support for the null hypothesis (no group difference)

We conducted the same analysis between recAN^−^ and the full HC^−^ sample (before age-matching), which yielded similar results (Supplemental Table 4). In short, there were no robust group differences in any of the blood parameters, which was confirmed by Bayesian statistics for glucose, insulin, GIP, and glicentin, but not for GLP-1 and glucagon.

#### OC^+^ groups

The original values of the blood parameter concentrations of the OC^+^ groups are presented in Table 4 alongside group comparison results after log_10_-transformation. Between these groups, none of the blood parameters exhibited significant differences after FDR correction. This was confirmed by Bayesian statistics for all parameters: glucose (BF_01_=4.121), insulin (*BF*_*01*_=2.535), GIP (*BF*_*01*_=4.192), GLP-1 (*BF*_*01*_=4.206), glicentin (*BF*_*01*_=4.072), and glucagon (*BF*_*01*_=3.665).

**Table 4:** Groups with oral contraceptive use (OC^+^): Blood glucose and hormone concentrations

	*HC*^*+*^ (*N* = 35)	*recAN*^*+*^ (*N* = 45)	Student’s T-test	FDR	
	Mean ± SD Median (IQR)	Mean ± SD Median (IQR)	*p*	*p*	BF_01_
**Glucose (mmol/l)**	5.03 ± 0.38	5.06 ± 0.41	0.771	0.85	4.121
**Insulin (pM)**	31.64 (23.03)	31.67 (15.55)	0.273	0.85	2.535
**GIP (pM)**	3.82 (3.43)	3.5 (2.05)	0.831	0.85	4.192
**GLP-1 (pM)**	4.28 (2.47)	3.72 (2.24)	0.850	0.85	4.206
**Glicentin (pM)**	32.97 (25.76)	32.19 (32.75)	0.733	0.85	2.072
**Glucagon (pM)**	3.47 (3.1)	3.4 (2.66)	0.563	0.85	3.665

Note. GIP=glucose-dependent insulinotropic polypeptide; GLP-1 = glucagon-like peptide-1 (GLP-1); HC=healthy controls; mmol/l= millimoles per litre; pM=picomolar; recAN=long-term recovered anorexia nervosa patients. Mean±standard deviation is reported for normally distributed parameters; median (interquartile range) is provided for non-normally distributed data. GIP, GLP-1, glicentin, and insulin concentrations were log₁₀-transformed prior to group comparisons. *P*-values were adjusted for multiple comparisons using the False Discovery Rate (FDR) correction method [49]. Asterisks indicate statistical significance (two-tailed): *p*<.01 (**), *p*<.05 (*). Bayes factors (*BF₀₁*) > 1 indicate increasing support for the null hypothesis (no group difference)

## Discussion

In this cross-sectional study, we investigated fasting serum concentrations of four gut-secreted proglucagon-derived hormones glucagon-like peptide-1 (GLP-1), glucose-dependent insulinotropic polypeptide (GIP), glucagon, and glicentin in individuals long-term recovered from anorexia nervosa (recAN), comparing them to healthy controls (HC) and stratifying by oral contraceptive (OC) use. To our knowledge, this is the first study to assess these hormones collectively in a recAN cohort. Our findings show no significant differences in hormone concentrations between recAN and HC, a pattern that was consistent across all hormones and independent of OC use, as confirmed by Bayesian statistics. In comparison, we found alterations in three (GIP, GLP-1, glicentin) out of the four hormones in individuals during either the acute phase of AN or after short-term weight recovery in a separate cohort in our previous study [32]. Taken together with our current findings, these alterations are likely to reflect a state-dependent response to undernutrition, i.e. they do not seem to persist after long-term recovery.

This interpretation is substantiated by evidence from a separate cohort showing that GIP levels are elevated in acute AN but normalize after short-term weight restoration, whereas GLP-1 and glicentin decreased during partial weight recovery [32]. Glucagon levels remained unchanged throughout. The current findings extend this trajectory by demonstrating that all four hormones show levels comparable to HC in recAN, reinforcing the idea that these changes are reversible and possibly reflect adaptive responses to undernutrition rather than enduring trait markers. Notably, the duration of recovery in recAN had no influence on any of the blood parameters (Supplemental Table 6). Comparison with findings at the opposite end of the weight spectrum provides further insights into the state-dependent nature of these alterations (Supplemental Fig. 1). In obesity, GIP concentrations do not differ from HC [53, 54], while findings for GLP-1 remain inconclusive [55–58]. Glicentin appears to be reduced or normal in individuals with obesity [55, 59], mirroring the pattern observed in patients with AN after short-term weight restoration [32]. Notably, glucagon levels are elevated in obesity [55, 60], contrasting with our findings of reduced or unchanged glucagon in both acute AN and following short-term weight restoration. Although the metabolic context differs between these conditions, alterations at opposite ends of the weight spectrum, paired with blood concentrations comparable to HC after recovery in AN, support the interpretation that alterations in gut-secreted hormones are linked to metabolic state rather than representing trait-based dysregulation.

Mechanistically, the proglucagon-derived hormones play a central role in regulating appetite, energy balance, and glucose homeostasis through both peripheral and central pathways. GLP-1 and GIP are incretin hormones that enhance insulin secretion in a glucose-dependent manner and modulate appetite through central and gastrointestinal mechanisms [13], with GLP-1 slowing gastric emptying and promoting satiety [61]. Glicentin, though less well-characterized, appears to regulate gastrointestinal motility and appetite [18], while glucagon, beyond its role in hepatic glucose production, may contribute to appetite suppression and energy expenditure, indicating a more nuanced metabolic function [62]. The fact that these hormones were within the range observed in HC in individuals recovered from anorexia nervosa suggests that their dysregulation in the acute phase of AN is likely a consequence of severe energy deficiency rather than a stable trait or predisposing factor. This supports the idea that gut hormone profiles are highly state-dependent and responsive to nutritional status. However, our cross-sectional design means we cannot directly link the absence of differences in hormone levels we observed relative to HC to a specific point in the recovery trajectory. Without repeated measurements during the acute phase, weight restoration, and long-term recovery within the same individuals, we cannot determine when exactly hormone levels reach those of HC or how gradually or abruptly this occurs or if there is some fluctuation. Notably, though, our previous longitudinal work found that these same appetite-regulating hormones normalize with short-term weight restoration following refeeding [32]. These findings suggest that convergence toward HC levels may occur relatively early in the recovery process. Even though these hormones are unlikely to serve as trait vulnerability markers, their dynamic changes across illness stages (Supplemental Fig. 1) raise the possibility that they could function as AN stage-specific biomarkers. For example, elevated GIP in acute AN may reflect altered nutrient sensing or compensatory mechanisms during starvation. Alternatively, such changes might not only represent adaptive responses but could potentially reflect mechanisms that contribute to the maintenance of the disorder. For instance, higher GIP levels could help maintain metabolic states that reinforce the restrictive eating behaviours through its stimulation of glucagon. If confirmed, such patterns could help delineate stages of illness and recovery, offering objective markers to complement clinical assessments. Nevertheless, this application remains speculative and requires longitudinal validation. In addition, it remains unknown how rapidly GIP responds to refeeding or whether it reflects food intake prior to weight gain during AN recovery. Future studies should assess postprandial hormone dynamics and explore whether GIP levels change before measurable weight restoration occurs.

Oral contraceptive use, while not influencing group differences in hormone levels, may still modulate metabolic parameters. Our supplementary analyses (Supplemental Material, 2.3.2. HC groups) suggest age-related effects on GIP, GLP-1, and glucagon, which could confound interpretation. Further, while Bayesian statistics confirmed that all examined blood parameters except glucagon did not differ between recAN and HC independent of OC use, more conflicting evidence on the influence of OC emerged from the comparison of our HC groups (Supplemental Table 5). The metabolic impact of OC use is known to vary by formulation and dosage [35, 36], and future studies should stratify by OC type and consider menstrual cycle phase to refine hormonal assessments.

Our findings also shed insight regarding the endocrine recovery in AN. We have shown in our previous longitudinal study that, in AN, these same appetite-regulating hormones normalize with short-term weight restoration following refeeding [32]. Our current findings extend this by showing that, several years into sustained recovery, proglucagon-derived gut hormones remain indistinguishable from those of HCs, suggesting that the alterations observed during the acute phase reflect a reversible, state-dependent response to undernutrition rather than a persistent endocrine trait of the disorder. Other metabolic hormones, such as leptin and ghrelin, have been extensively studied in this context [9, 10]. Indeed, the ratio between circulating levels of ghrelin and Liver Enriched Antimicrobial Peptide 2 (LEAP2, an endogenous antagonist for the ghrelin receptor) has been proposed as a metabolic recovery biomarker [63]. However, levels of the gut hormones GIP, GLP-1, glicentin, and glucagon in AN staging remain underexplored. To our knowledge, they have not previously been examined this far into sustained recovery. Our results suggest that, in this cross-sectional sample, gut hormone concentrations after sustained recovery from AN were indistinguishable from those of HC. Whether gut hormone profiles could eventually serve as a marker of endocrine recovery status, or whether deviations from typical HC levels might be informative for identifying incomplete recovery, remains speculative and would require further longitudinal investigation to establish. Our cross-sectional findings cannot, on their own, support conclusions about relapse risk or recovery trajectories at the individual level.

Animal studies and research in obesity further underscore the plasticity of gut hormone systems in response to metabolic states. For example, GLP-1 receptor agonists are widely used in the treatment of obesity and type 2 diabetes, reflecting their pivotal role in appetite regulation and energy homeostasis [61]. Notably, while individuals with obesity often exhibit persistent gut hormone dysregulation even after weight loss, our findings in recAN suggest a restoration of hormonal balance (Supplemental Fig. 1). This contrast highlights the state-dependent nature of gut hormone adaptations, with recAN potentially representing a return to homeostasis rather than a lingering pathological profile.

### Limitations

Several limitations should be noted. First, the cross-sectional design prevents conclusions about the time course of hormonal normalization. While our sample includes individuals who have recovered from AN for several years, we cannot directly track hormonal trajectories from the acute stage through weight restoration to long-term recovery in the same participants. Longitudinal studies following individuals with AN across those stages would provide stronger evidence regarding the pace and consistency of a hormonal normalization process. Second, we assessed hormones only in the fasting state. GLP-1 and GIP are incretins whose primary role is the regulation of postprandial glucose metabolism and satiety signalling [13, 14]. Fasting measurements capture only baseline secretion profiles and cannot reveal potential differences in endocrine responses to nutrient intake. Such postprandial dynamics or subtle regulatory shifts could persist after recovery even in the absence of fasting differences and would not be detected by our design. Standardized meal challenges and postprandial time-series sampling would be needed to characterise the functional regulation of these hormones more fully and to strengthen mechanistic interpretation of our findings. Third, while OC use was controlled for, its metabolic effects are complex and may interact with age, hormonal profiles, and recovery status. Finally, our recAN and HC sample consisted exclusively of females, limiting generalizability to males with AN.

## Conclusion

This study provides the first collective investigation of GLP-1, GIP, glicentin, and glucagon in recAN. In summary, our findings demonstrate that concentrations of gut-secreted proglucagon-derived hormones involved in appetite and glucose regulation do not differ between individuals long-term recovered from AN and HC. These results support a state-dependent interpretation of hormonal alterations in AN and suggest that the gut endocrine system is capable of full recovery. While these hormones are unlikely to serve as trait markers, their dynamic changes across illness stages may offer insights into physiological recovery and potential biomarkers for treatment monitoring. Future longitudinal and mechanistic studies are needed to validate these findings and explore their clinical utility.

## Supplementary information

Below is the link to the electronic supplementary material.

Supplementary Material 1

## Data Availability

The datasets used and/or analysed during the current study are available from the corresponding author on reasonable request.

## Abbreviations

AN: anorexia nervosa
BDI-II: Beck-Depression-Inventar Revision
BMI: body mass index
BMI-SDS: body mass index standard deviation score
EDI-2: Eating Disorder Inventory-2
FDR: false discovery rate
GIP: gastric inhibitory peptide
GIPR: drug-induced gastric inhibitory peptide receptor
GLP-1: glucagon-like peptide-1
HC: healthy controls
HC^+^: healthy controls with oral contraceptive use
HC^−^: healthy controls without oral contraceptive use
IQ: intelligence quotient
OC: oral contraceptive
OC^+^: with oral contraceptive use
OC^−^: without oral contraceptive use
recAN: individuals who are long-term recovered from AN
recAN^+^: individuals who are long-term recovered from AN with oral contraceptive use
recAN^−^: individuals who are long-term recovered from AN without oral contraceptive use
SCL-90-R: Symptom-Checklist-90 Revised
SIAB-EX: Structured Interview for Anorexia and Bulimia Nervosa
