# Supplementary material for: Fasting gut hormone levels following long-term recovery from Anorexia Nervosa: A cross-sectional study
Source: J Eat Disord. 2026 Sep 10;14:217. doi: 10.1186/s40337-026-01763-y (PMC13563803; doi:10.1186/s40337-026-01763-y)
Supplement: Supplementary file 1 [file 40337_2026_1763_MOESM1_ESM.docx]

*Supplemental Materials:* Normalization of Gut Hormone Levels Following Long-Term Recovery from Anorexia Nervosa

Theresa Kolb^1^, Friederike I. Tam^1^, Claudia Förster Ribet^1^, Michaela Ohme^1^, Lina C. Schlolaut^1^, Joseph A. King^1^, Veit Rössner^2^, Suzanne L. Dickson^3^, Alessandra Borsini^4^, Nikolaos Perakakis^5,7,8,§^, Stefan Ehrlich^1,6,§^

^1^Division of Psychological and Social Medicine and Developmental Neurosciences, Faculty of Medicine, Technische Universität Dresden, Dresden, Germany;

^2^Department of Child and Adolescent Psychiatry and Psychotherapy;

^3^Institute of Neuroscience and Physiology, The Sahlgrenska Academy at the University of Gothenburg Sweden;

^4^Stress, Psychiatry and Immunology Laboratory, Institute of Psychiatry, Psychology and Neuroscience, Department of Psychological Medicine, King's College London, London, UK;

^5^Department of Internal Medicine III, University Hospital Carl Gustav Carus, Technische Universität Dresden;

^6^Eating Disorder Treatment and Research Center, Department of Child and Adolescent Psychiatry, Faculty of Medicine, Technische Universität Dresden, Dresden, Germany;

^7^Paul Langerhans Institute Dresden (PLID), Helmholtz Center Munich, University Hospital and Faculty of Medicine;

^8^German Center for Diabetes Research (DZD e.V.), Neuherberg, Germany;

^§^Both authors contributed equally to this work.

## 1. Methods

## 1.1. *Age matching*

Due to significant age differences between the healthy control (HC) group (M=16.90, SD=2.72) and the recovered anorexia nervosa (recAN) group (M=22.57, SD=3.85), as well as an imbalance in sample sizes (HC: N=65; recAN: N=35), we applied an age-matching procedure using the *MatchIt* package in R to improve group comparability. Participants were matched in a 1:1 ratio based on age, and intra-pair age differences were calculated by subtracting the age of each HC participant from that of their matched recAN counterpart. Following matching, the mean age difference between pairs was 4.51 years, with a maximum difference of 13.10 years. The mean age of the HC group increased slightly after matching (M=18.33, SD=2.99), yet a significant residual age difference between the groups remained. Therefore, age was included as a covariate in all subsequent analyses involving these subgroups.

## 2. Results

Participants of both groups (recAN, HC) were further stratified into groups based on oral contraceptive (OC) use, coded as "^+^" (OC users) or "^−^" (non-users).

## 2.1. *Demographic, anthropometric and symptom variables*

### 2.1.1. OC^−^ groups

RecAN^−^ and HC^−^ differed significantly in parameters of psychopathology (BDI-II, EDI-2, SCL-90-R) (Supplemental Table 1). Because of the persistent age differences even after age-matching we conducted ANCOVAs to control for age. Differences in psychopathology remained significant.

**Supplemental Table 1.** OC^−^ group: Parameters of psychopathology.

|  | *HC*^−^ | | *recAN*^−^ | | ***p*** |
| --- | --- | --- | --- | --- | --- |
|  | **N** | **Median (IQR)** | **N** | **Median (IQR)** |  |
| **BDI-II** | 32 | 3.00 (5.00) | 33 | 9 (13.00) | .002** |
| **EDI-2** | 32 | 134.50 (38.25) | 34 | 169.5 (57.41) | <.001** |
| **SCL-90-R** | 32 | 22.50 (22.00) | 34 | 7.1 (4.89) | .035* |

*Note.* BDI-II=Beck Depression Inventory-II; EDI-2=Eating Disorder Inventory-2; HC=healthy controls; recAN=long-term recovered anorexia nervosa patients; SCL-90-R=Symptom Checklist-90-Revised. Median (interquartile range) is reported for non-normally distributed parameters. Group comparisons were performed using ANCOVA with age as a covariate. Asterisks indicate statistical significance (two-tailed): *p*<.01 (**), *p*<.05 (*).

### 2.1.2. HC groups

HC^+^ and HC^−^ showed significant differences in parameters of psychopathology (BDI-II, EDI-2, SCL-90-R) (Supplemental Table 2). Due to the age differences, we conducted additional ANCOVAs for these parameters. After controlling for age, there was no difference in psychopathology between the groups (Supplemental Table 3).

**Supplemental Table 2**. HC groups before age-matching: Demographic and clinical characteristics.

|  | *HC*^−^ | | *HC^+^* | | *Group Comparison* |
| --- | --- | --- | --- | --- | --- |
|  | **N** | **Mean±SD**  **Median (IQR)** | **N** | **Mean±SD**  **Median (IQR)** | **Uncorrected *p*-value** |
| **Age** | 65 | 16.30 (2.20) | 35 | 22.90 (4.45) | <.001** |
| **BMI (kg/m^2^)** | 65 | 21.00±2.14 | 35 | 21.92±2.43 | .064 |
| **BMI-SDS** | 65 | 0.00±0.74 | 35 | -0.18±0.68 | .240 |
| **Minimal Lifetime BMI (kg/m^2^)** | 56 | 20.18±1.85 | 33 | 20.11±1.68 | .837 |
| **IQ** | 64 | 112.03±9.94 | 35 | 110.23±9.56 | .379 |
| **BDI-II** | 61 | 4.00 (6.00) | 29 | 1.00 (3.00) | .001** |
| **EDI-2** | 61 | 141.00 (41.00) | 28 | 124.5 (22.74) | .015* |
| **SCL-90-R** | 61 | 20.00 (25.00) | 28 | 9.00 (12.75) | .004** |
| **Leptin (ng/ml)** | 61 | 8.98 (9.53) | 29 | 11.86 (11.60) | .075 |

*Note.* BDI-II=Beck Depression Inventory-II; BMI=body mass index; BMI-SDS=body mass index standard deviation score; EDI-2=Eating Disorder Inventory-2; HC=healthy controls; IQ=intelligence quotient; kg/m²=kilograms per square meter; ng/ml=nanograms per millilitre; SCL-90-R=Symptom Checklist-90-Revised. Mean±standard deviation is reported for normally distributed parameters; median (interquartile range) is reported for non-normally distributed parameters. Group comparisons were performed using Student’s t-test or Mann–Whitney U test, as appropriate. Asterisks indicate statistical significance (two-tailed): *p*<.01 (**), *p*<.05 (*).

**Supplemental Table 3.** HC groups after age-matching: Parameters of psychopathology.

|  | *HC*^−^ | | *HC^+^* | | ***p*** |
| --- | --- | --- | --- | --- | --- |
|  | **N** | **Mean±SD**  **Median (IQR)** | **N** | **Mean±SD**  **Median (IQR)** |  |
| **BDI-II** | 61 | 4.00 (6.00) | 29 | 1.00 (3.00) | .156 |
| **EDI-2** | 61 | 141.00 (41.00) | 28 | 124.50 (22.74) | .272 |
| **SCL-90-R** | 61 | 20.00 (25.00) | 28 | 9.00 (12.75) | .349 |

*Note*. BDI-II=Beck Depression Inventory-II; EDI-2=Eating Disorder Inventory-2; HC=healthy controls; SCL-90-R=Symptom Checklist-90-Revised. Median (interquartile range) is reported for non-normally distributed parameters. Group comparisons were performed using ANCOVA with age as a covariate. Asterisks indicate statistical significance (two-tailed): *p*<.01 (**), *p*<.05 (*).

### 2.2. Blood glucose and hormone concentrations

### 2.2.1. OC^−^ groups

**Supplemental Table 4**. Supplementary group comparisons of OC^−^ groups before age-matching.

|  | *HC*^−^ *(N=65)* | *recAN*^−^ *(N=35)* | *ANCOVA* | *FDR* | ***BF_01_*** |
| --- | --- | --- | --- | --- | --- |
|  | **Mean±SD**  **Median (IQR)** | **Mean±SD**  **Median (IQR)** | ***p*** | ***p*** |  |
| **Glucose (mmol/l)** | 5.12±0.44 | 5.01±0.34 | .949 | .949 | 2.691 |
| **Insulin (pM)** | 35.90 (20.56) | 28.44 (13.62) | .393 | .786 | 92.255 |
| **GIP (pM)** | 5.34 (3.79) | 4.81 (3.31) | .758 | .916 | 4.438 |
| **GLP-1 (pM)** | 3.46 (1.53) | 4.08 (1.93) | .192 | .576 | 1.000 |
| **Glicentin (pM)** | 27.59 (18.92) | 28.44 (24.03) | .763 | .916 | 2.493 |
| **Glucagon (pM)** | 4.52 (2.76) | 3.69 (2.44) | .078 | .468 | 1.000 |

*Note.* ­GIP=glucose-dependent insulinotropic polypeptide; GLP-1= glucagon-like peptide-1; HC=healthy controls; mmol/l= millimoles per litre; pM=picomolar; OC=oral contraceptive; recAN=long-term recovered anorexia nervosa patients. Mean±standard deviation is reported for normally distributed parameters; median (interquartile range) is provided for non-normally distributed data. GIP, GLP-1, glicentin, and insulin concentrations were log₁₀-transformed prior to group comparisons. ANCOVAs were conducted with age as a covariate. Asterisks indicate statistical significance (two-tailed): *p*<.01 (**), p<.05 (*). *P*-values were adjusted for multiple comparisons using the False Discovery Rate (FDR) correction method (1)**. Bayes factors (*BF₀₁*)>1 indicate increasing support for the null hypothesis (no group difference)**.

### 2.2.2. HC groups after age-matching

As part of a supplementary analysis, we compared blood parameter concentrations of the HC groups (Supplemental Table 5). No significant differences were found for any of the parameters after controlling for age and correcting for multiple comparisons (FDR). Bayesian analysis confirmed that there is no difference in glucose (*BF_01_*=3.392), insulin (*BF_01_*=68.340), and GLP-1 (*BF_01_*=1.532), but not in GIP (*BF_01_*=1.000), glicentin (*BF_01_*=1.000) and glucagon (*BF_01_*=1.000). Without age correction, GIP and glucagon levels were decreased in HC^+^, while glicentin was elevated.

**Supplemental Table 5**. HC groups after age-matching: Blood glucose and hormone concentrations.

|  | *HC*^−^ *(N=65)* | *HC^+^ (N=35)* | *ANCOVA* | *FDR* | ***BF_01_*** |
| --- | --- | --- | --- | --- | --- |
|  | **Mean±SD**  **Median (IQR)** | **Mean±SD**  **Median (IQR)** | ***p*** | ***p*** |  |
| **Glucose (mmol/l)** | 5.1±0.44 | 5.03±0.38 | .966 | .966 | 3.392 |
| **Insulin (pM)** | 35.9 (20.56) | 31.64 (23.03) | .023* | .138 | 68.340 |
| **GIP (pM)** | 5.34 (3.79) | 3.82 (3.43) | .053 | .154 | 1.000 |
| **GLP-1 (pM)** | 3.46 (1.53) | 4.28 (2.47) | .628 | .754 | 1.532 |
| **Glicentin (pM)** | 27.59 (18.92) | 32.97 (25.76) | .302 | .453 | 1.000 |
| **Glucagon (pM)** | 4.52 (2.76) | 3.47 (3.1) | .077 | .154 | 1.000 |

*Note.* ­GIP=glucose-dependent insulinotropic polypeptide; GLP-1= glucagon-like peptide-1; mmol/l= millimoles per litre; pM=picomolar; recAN=long-term recovered anorexia nervosa patients; HC=healthy controls. Group “−” does not use oral contraceptives (OC); group “+” uses OC. Mean±standard deviation is reported for normally distributed parameters; median (interquartile range) is provided for non-normally distributed data. GIP, GLP-1, glicentin, and insulin concentrations were log₁₀-transformed prior to group comparisons. ANCOVAs were conducted with age as a covariate. Asterisks indicate statistical significance (two-tailed): *p*<.01 (**), *p*<.05 (*). *P*-values were adjusted for multiple comparisons using the False Discovery Rate (FDR) correction method (1)**. Bayes factors (*BF₀₁*)>1 indicate increasing support for the null hypothesis (no group difference)**.

### 2.3.3 RecAN^−^ group

No significant correlations were found between blood parameters and time since recovery (in months) in the recAN^−^ group.

***Supplemental Table 6.*** *RecAN^−^ group: Pearson’s correlations of the concentrations of blood glucose and hormone concentrations and time since recovery.*

|  | **Glucose (mmol/l)** | **Insulin (pM)** | **GIP (pM)** | **GLP-1 (pM)** | **Glicentin (pM)** | **Glucagon (pM)** |
| --- | --- | --- | --- | --- | --- | --- |
| **Months since recovery** | 0.051 | -0.229 | 0.070 | -0.018 | 0.115 | 0.067 |

*Note.* GIP=glucose-dependent insulinotropic polypeptide; GLP-1= glucagon-like peptide-1; mmol/l= millimoles per litre; pM=picomolar; recAN=long-term recovered anorexia nervosa patients. Pearson’s r reported for the correlations between blood glucose and hormone concentrations, and months since recovery from anorexia nervosa. GIP, GLP-1, glicentin, and insulin concentrations were log₁₀-transformed prior to analysis. Asterisks indicate statistical significance (two-tailed): *p*<.01 (**), *p*<.05 (*).

## 3. Discussion

**Supplemental Figure 1**. Alterations in fasting circulating gut hormone concentrations across metabolic and diagnostic groups.

Schematic representation of alterations in fasting circulating gut hormone concentrations across metabolic groups from our previous (acAN-T1=patients with anorexia nervosa (AN) in the acute state; acAN-T2=patients with AN after short-term weight restoration) and current study (recAN^−^=recovered AN without oral contraceptive (OC) use; recAN^+^=recovered AN with OC use), as well as literature on obesity (2–9). Differences are illustrated using coloured equals signs and directional arrows, with arrow orientation (upward or downward) indicating increased or decreased concentrations relative to the specified comparator group. Colour coding denotes the reference group for comparison: green=healthy controls (HC), orange=acAN-T1, light green=HC without OC, dark green=HC with OC, and yellow=comparisons with HC reported in the literature. ­GIP=glucose-dependent insulinotropic polypeptide; GLP-1= glucagon-like peptide-1.

## 4. References

1.	Benjamini Y, Hochberg Y. Controlling the False Discovery Rate: A Practical and Powerful Approach to Multiple Testing. Journal of the Royal Statistical Society: Series B (Methodological). Januar 1995;57(1):289–300.

2.	Elahi D, Andersen DK, Muller DC, Tobin JD, Brown JC, Andres R. The Enteric Enhancement of Glucose-stimulated Insulin Release: The Role of GIP in Aging, Obesity, and Non-insulin-dependent Diabetes Mellitus. Diabetes. 1. Oktober 1984;33(10):950–7.

3.	Greenfield JR, Farooqi IS, Keogh JM, Henning E, Habib AM, Blackwood A, u. a. Oral glutamine increases circulating glucagon-like peptide 1, glucagon, and insulin concentrations in lean, obese, and type 2 diabetic subjects. The American Journal of Clinical Nutrition. Januar 2009;89(1):106–13.

4.	Heptulla RA, Tamborlane WV, Cavaghan M, Bronson M, Limb C, Yong-Zhan MA, u. a. Augmentation of Alimentary Insulin Secretion despite Similar Gastric Inhibitory Peptide (GIP) Responses in Juvenile Obesity. Pediatr Res. Mai 2000;47(5):628–33.

5.	Lugari R, Dei Cas A, Ugolotti D, Camellini C, Ganzerla A, Luciani A, u. a. Glucagon-like Peptide 1 (GLP-1) Secretion and Plasma Dipeptidyl Peptidase IV (DPP-IV) Activity in Morbidly Obese Patients Undergoing Biliopancreatic Diversion. Horm Metab Res. Februar 2004;36(2):111–5.

6.	Manell H, Staaf J, Manukyan L, Kristinsson H, Cen J, Stenlid R, u. a. Altered Plasma Levels of Glucagon, GLP-1 and Glicentin During OGTT in Adolescents With Obesity and Type 2 Diabetes. The Journal of Clinical Endocrinology & Metabolism. 1. März 2016;101(3):1181–9.

7.	Raffort J, Panaïa-Ferrari P, Lareyre F, Blois M, Bayer P, Staccini P, u. a. Decreased serum glicentin concentration in patients with severe and morbid obesity. Ann Clin Biochem. März 2018;55(2):198–204.

8.	Stern JH, Smith GI, Chen S, Unger RH, Klein S, Scherer PE. Obesity dysregulates fasting-induced changes in glucagon secretion. Journal of Endocrinology. November 2019;243(2):149–60.

9.	Stinson SE, Jonsson AE, Lund MAV, Frithioff-Bøjsøe C, Aas Holm L, Pedersen O, u. a. Fasting Plasma GLP-1 Is Associated With Overweight/Obesity and Cardiometabolic Risk Factors in Children and Adolescents. The Journal of Clinical Endocrinology & Metabolism. 13. Mai 2021;106(6):1718–27.
